# Exploring factors influencing the uptake of kangaroo mother care: key informant interviews with parents

**DOI:** 10.1186/s12884-023-06021-6

**Published:** 2023-10-03

**Authors:** Wai Cheng Foong, Siew Cheng Foong, Jacqueline J. Ho, Divya Gautam, Jen Jen Leong, Pek Yong Tan, Mehala Baskaran

**Affiliations:** 1 Department of Paediatrics, RCSI & UCD Malaysia Campus, George Town, Penang Malaysia; 2 Department of Obstetrics and Gynaecology, University Hospital Galway, Galway, Ireland; 3 Department of Paediatrics, Sunway Medical Centre, Perai, Penang Malaysia; 4 Department of Paediatrics, Island Hospital, George Town, Penang Malaysia; 5 Department of Paediatrics, Pulau Pinang Hospital, Ministry of Health, George Town, Penang Malaysia

**Keywords:** Kangaroo mother care, Breastfeeding, Preterm, Skin-to-skin, Triandis, Infants, Parenthood, Discharge, Weight gain, Barrier

## Abstract

**Background:**

The updated World Health Organization (WHO) guideline recommends immediate kangaroo mother care (KMC) for all infants, particularly those born preterm. However, its uptake and sustainability have been unsatisfactory. Therefore, we aimed to gain deeper insight into factors influencing the uptake of KMC practice in our setting, and thereby identify possible solutions for the development of relevant interventions to improve its adoption and make KMC a routine practice for all infants.

**Methods:**

Using the Triandis model of social behaviour as our framework, we conducted key informant interviews with parents and healthcare providers. Trained interviewers conducted interviews with nine parents, recruited via purposive sampling. These parents were parents of preterm infants who had been introduced to KMC. Data was transcribed and analysed based on Triandis’ Theory of Interpersonal Behaviour. This paper only reports the results of the parent interviews.

**Results:**

Major findings were how positive feelings like warmth and contentment, the sense of parenthood with KMC, the benefits of KMC for their infant and parents being enablers for KMC uptake. Conversely, the lack of KMC awareness, the initial negative feelings such as fear, uncertainty and embarrassment, the prioritization of time for milk expression, overcrowding in the ward, lack of space and privacy, limited visiting hours, lack of support and poor communication resulting in misapprehension about KMC were major barriers.

**Conclusion:**

A deeper understanding of the factors influencing the uptake of KMC using the Triandis behavioural model provided a way forward to help improve its uptake and sustainability in our settings.

**Trial registration:**

This study was registered with the National Medical Research Registry (NMRR-17-2984-39191).

**Supplementary Information:**

The online version contains supplementary material available at 10.1186/s12884-023-06021-6.

## Background

“Kangaroo Mother Care” (KMC) is a method of care for preterm infants and breastfeeding support. It is often confused with ‘skin-to-skin contact’ that babies get within first hour of life at the labour room. KMC is defined as prolonged periods of skin-to-skin contact between a baby and an adult beyond the skin-to-skin contact in the labour room. The adult is usually the baby’s mother, but the person can be the father, older siblings, adult family members or friends [1]. It is endorsed by the International Paediatric Association (IPA) and World Health Organization (WHO) as a key to low birth weight and or preterm infants’ survival in all settings [2–6]. Numerous studies have shown that KMC implementation is bounded by many factors such as awareness and acceptance, readiness, socio-cultural settings, hospital policies, advocacy for KMC and an individual’s intention to perform KMC [7–9].

In Malaysia, although skin-to-skin at the first hour of life is recommended as standard practice, KMC is only practised by some individual neonatal units, and has yet to be incorporated into standard care for newborns. There is little information, if any, in Malaysia about the extent of its implementation. Comments within the local neonatal community suggest that KMC implementation is widely variable. Indeed, it is our experience that in spite of our structured education programme through the SEA-URCHIN project (South East Asia-Using Research for Change in Neonatal Infection) in 2013 [10], KMC implementation at full scale at our participating hospitals has been difficult.

One of the difficulties faced when promoting the development of KMC as a norm for all preterm infants was the lack of sufficient information concerning important determinants of this practice in our population. Therefore, we aimed to gain deeper insight into factors influencing the uptake of KMC practice in our setting, and thereby identify possible solutions for the development of relevant interventions to improve its adoption and make KMC routine practice for all infants. Using the Triandis’ Theory of Interpersonal Behaviour as our framework, we conducted key informant interviews with parents and healthcare providers. This paper reports the parent interviews.

Please note that during the period of this study, the recommendation was that KMC be the norm for preterm infants. In November 2022 after the study was over, this recommendation was updated and the WHO’s guidelines now state that KMC is to be norm for all infants [4].

## Materials & methods

### Study setting

Key informant interviews were conducted between April 2018 to September 2018 at Penang Hospital and Seberang Jaya Hospital. Both are public hospitals in Penang, Malaysia. Healthcare staff in the neonatal unit and the obstetric unit from both hospitals were formally trained to support KMC practices in 2013 through the SEA-URCHIN project (South East Asia-Using Research for Change in Neonatal Infection) which used an evidence-based skill teaching method [10, 11]. The training was initiated following the WHO guidelines in 2003 for stable preterm or low-birth-weight babies [3].

The nursing staff in these units were all female but there were male and female doctors. All of them had been trained to identify babies who were eligible for KMC. (At that point in time, KMC was recommended only for infants that were clinically stable). They had also been trained on how to provide hands-on assistance to parents initiating KMC. This included how to correctly place the baby skin-to-skin in a frog-like position onto the chest of the parent and how to secure the baby using a binder so that the parent could move about freely. Of note, there are many types of binders that can be used to secure the baby to the parent, and in our setting, we used a tube made of elasticated cloth as the binder. Staff had also been trained to identify parents who could position the baby for KMC by themselves without assistance from healthcare staff, and how to monitor the baby while practicing KMC.

In contrast to the current recommendation, at the time of the study, KMC was only implemented for preterm infants who did not require intensive care and for those who had been moved out of the intensive care unit. Therefore, KMC was performed only on stable babies with the approval of a senior doctor and only when there were healthcare staff available for regular monitoring of the baby. Hence, it was generally performed beside the baby’s cot or incubator in the open and often crowded ward. Privacy was provided using portable screens. One of the hospitals had in addition created a small KMC corner which consisted of a curtained enclosure in the corner of the ward. This corner provided privacy for KMC practice and for mothers to breastfeed. In Malaysia, Muslim women generally completely cover up revealing only their faces and hands and some prefer to cover their faces as well. A private space to perform KMC was therefore essential to them.

The availability of beds for mothers to stay overnight (rooming-in facilities) with their babies was limited in both hospitals. Only one of the two hospitals allowed mothers to perform KMC in the rooming-in area because there was limited supervision by healthcare staff in these rooms. Where appropriate, the mother was given the responsibility to care for and monitor her baby throughout the process.

### Recruitment of informants

The inclusion criteria in this study were parents of stable preterm infants (born before 37 completed weeks of gestation) or low birth weight infants (less than 2500 g) and those who had prior KMC introduction or KMC experience. Using purposive sampling, mothers or fathers who fulfilled the inclusion criteria were approached by a senior doctor who then gave information about the study. After obtaining initial consent from the participant, the senior doctor would coordinate with the trained interviewers on the date, time, and venue to conduct the interview. Just before commencing each interview, the trained interviewers would reconfirm the participant’s willingness to be interviewed and obtain written consent. Our plan was to continue recruiting participants until the interviews did not reveal any new information.

### Interviews

To reduce variability, we collaboratively developed a set of semi-structured open-ended questions. We discussed among ourselves on how to elicit emotional, social and behavioural elements of parents’ experiences and intention to practise KMC. An initial set of questions were drafted based on this discussion, and we subjected it to a pretesting phase with some parents and healthcare workers whose results were not included as part of this study’s results. This pretest served both to identify and address potential issues with the question, and to provide initial training for our interviewers. Feedback from the pretest led to necessary refinements to the questions as well as how to ask additional probing questions to obtain further information when necessary. Some demographic questions were asked to provide context to the findings (Appendix 2 Table 1). The questions were made available in three major languages (Malay, English and Chinese). See Appendix 1. The non-English versions were cross-check for congruency in translation and accuracy of the English representation.

The interviews were conducted by any two of the three trained interviewers. All the interviewers were fluent in English and Malay and one of them was also fluent in Chinese. The interviewers underwent training on how to obtain information from participants without inadvertently influencing their responses through mock interviews and the insights gained from the pre-test interviews. In addition to noting down initial responses to the four semi-structured questions, our interviewers were trained to be on the alert for expressions, phrases and words that could suggest potentials to unveil further insights or details; and to use probing questions such as “Why do you feel like this …?”, “What do you mean by …?”, “What happened …?” and “How to …?” to expand on their initial responses and provide further details or clarification.

The interviews were conducted in Ward Sister’s room (a quiet room in the neonatal ward), using the preferred language of the participant (Malay, Chinese and or English). The interviewer fluent in the preferred language of the participant would conduct the interview and all interviews were recorded.

### Data and analysis

Two interviewers independently transcribed the interviews verbatim into a word processor from the audio recordings. They then compared their transcripts for any discrepancies between the audio recordings and the text. Each non-English transcript was later translated by one of the interviewers into English. All translated versions were checked by three of the investigators (PYT, JJL, MB) for congruency in translation and accuracy of the English representation.

Next, we applied the Braun and Clarke (2006) thematic analysis framework [12] to the data. WCF, SCF and DG first familiarised themselves with the data by reviewing the transcript while listening to the recording several times. This helped us be aware of the emotional ambience of the interviews: their hesitation, sighs, pride, etc. WCF, SCF and DG then organised the data into a meaningful and manageable way by generating initial codes. This was done by working through hard copies of the transcripts and marking relevant sections with pens and highlighters. Subsequently, WCF, SCF, DG and JJH examined the codes and searched for themes. These were then categorised and analysed using the Triandis’ Theory of Interpersonal Behaviour as our framework [13].

In some cases, we came across data that did not fit neatly into one variable and used consensus to decide where it would best fit or categorised it into more than one of the Triandis variables. These phases were not linear as we had to move forward and backwards between them several times.

## Results

### Description of participants

During the study period, there were only nine parents in the two study sites that fulfilled the inclusion criteria. All nine of them agreed to be interviewed. There were eight mothers and a father. Each interview took approximately 30 minutes to complete. The participants spoke mainly in their mother tongue (either Malay or Chinese) but it was common for some of them to insert English words and phrases as they were talking. Their infants’ gestation at birth ranged from 27 weeks to 33 weeks. The babies had been hospitalized between 15 and 69 days, and most of them had required at least two days of intensive care. All the participants had a KMC tube to facilitate KMC. See Appendix 2 Table 1.

Most participants practised KMC only once a day, and each KMC session was between 30 minutes to two hours in duration. Six participants had accommodation in the wards (roomed-in) and three commuted to the hospital daily from home.

### Interview results for each Triandis variable

#### The ‘habit’ component

We found that none of the participants would automatically have thought about practicing KMC in our hospital settings. This was because almost all of them had not heard about KMC before and were unaware that this was a form of newborn care. Several participants reported that an earlier introduction to the KMC concept would have better prepared them to do KMC when the opportunity arose.

*“They called me suddenly after 3 weeks, asking me to come and do KMC. Before this, none of the doctors and nurses said anything. When I came, I had no idea what I should do. I wasn’t even told what to wear.”* (Mother 1030).

Interventions for their baby such as the baby being in an incubator, the presence of the nasogastric tube, intravenous lines and other medical adjuncts added to their insecurity.

*“…because he was in the incubator. …., he must be critical. So, I shouldn’t disturb him.”* (Mother 1042).

The Chinese mothers also mentioned that it was important for them to have the traditional 4-week post-partum confinement practices that are commonly practiced by ethnic Chinese, and this included not going outside. Hence it was difficult to come to the hospital for KMC.

*“I feel like I am not close to the baby when he was admitted to the NICU, furthermore I was in the confinement centre (which was far from the hospital) at that time. So, it was very inconvenient for me. I was already expressing my breast milk from time to time. Otherwise, I will experience breast engorgement. I have to wash all the pumping utensils after that. I lack sleep after doing all these things. I’ll do this (KMC) after I get enough rest.”* (Mother 1103).

We found that, unlike breastfeeding, KMC was never part of social conversation in the wards. Participants talked about breastfeeding and its benefits, but did not mention KMC during conversations with each other. No one thought to encourage other parents to try KMC for their babies. We also found that no one mentioned how KMC could have improved breastfeeding for their baby during our interview.

#### The ‘intention’ components

##### Affect

All participants reported emotions ranging from feeling scared to being uncertain about whether they were capable enough to provide KMC safely to their baby especially when they were first told about it.

*“….putting his legs in this frog-like position… I am afraid it’ll be uncomfortable for him.”* (Father 1202).

*“I can’t! I have three babies. When they cry for feeds all at the same time, I panic … I don’t know what to do. I can’t bear seeing my babies cry… can’t imagine myself doing this (KMC).”* (Mother 1204 of a set of triplets).

*“When the nurses told me that I could start performing KMC, I felt so stressed about it that I went home straight away and did not come back for 3 days. There are too many things to do. I can’t do it by myself.”* (Mother 1028 said this as she looked at the busy ward).

They also felt it was stressful to have to monitor their baby’s well-being during KMC.

*“The nurses tell me to observe if my baby’s face has bluish or purplish discoloration. … I feel anxious and will be constantly observing his face, wondering if he is alright. It’s very stressful when I have to do this*.*”* (Mother 1916).

*“It’s because of the nurses here…First, I was taught how to do it (KMC). Next, the nurses expected me to master tube feeding after only teaching me once. I am still struggling with this (KMC). Why do I need to do all these? Why (is it) not (done) by the nurses?”* (Mother 1028).

Some participants felt shy, embarrassed and uneasy at the thought of doing KMC in an open ward. They felt that there were inadequate facilities to ensure privacy.

*“I feel exposed. … embarrassed. …. Yes, screens provide some privacy but I still don’t feel comfortable if there are men or even ladies if they were not of the Islamic faith on the other side of the screen.”* (Mother 1042).

All participants reported that they developed positive feelings towards KMC after they experienced it a few times. These feelings were expressed as feelings of warmth, contentment, happiness and excitement.

*“Our hearts beat in synchrony.”* (Mother 1444).

Some participants described having mixed positive and negative feelings while they performed KMC. For example, the father interviewed had conflicting emotions as he was keen to perform KMC, but he felt anxious about embarrassing the mothers who were present as well as the female staff who had to help him.

*“ …I don’t mind doing KMC. This is the only thing I can do for my baby. I have longed to hug him and KMC gave me the opportunity. These are enjoyable moments. It’s just that the ladies including the nurses may feel uncomfortable especially when I go into the (KMC) corner and sit with other mothers who are breastfeeding or doing KMC. I am also concerned that the nurses might feel embarrassed helping me to position the baby on my bare chest.”* (Father 1202).

Participants wished KMC had been introduced to them much earlier.

*“I feel exposed… he (baby) must be critical… (because he’s still in an incubator) … but then, when I held him so close, for the first time, I can see he’s so relaxed and slept so soundly on me…(paused followed with a sigh). What a pity. I should be told about this (KMC) before coming to this ward. I should have done this earlier.”* (Mother 1042).

Some participants reported that although they felt good doing KMC, they felt stressed with the heavy responsibility of monitoring the wellbeing of their still vulnerable baby or the thought of having to do KMC for at least six hours a day.

*“… he looks very peaceful, as if he feels safe. It’s such a warm feeling. …but when the nurses keep reminding me to check my baby’s colour, I can’t help keep thinking about how KMC might be dangerous.… The nurses also told me to do it (KMC) for at least six hours a day. … I just couldn’t comply. I don’t want to force myself … There should no pressure on me to do a fixed amount of time a day. Only then can KMC be more enjoyable.”* (Mother 1916).

Practicing KMC for long hours in the KMC corner made some participants feel isolated.

*“I feel bored doing it (KMC) by myself. Staying here is like staying in another world where you cannot meet your family.”* (Mother 1028).

##### Social

Participants felt that society expected them to do their best for their babies, therefore there was an obligation to do KMC because it was beneficial for their babies. It was an also opportunity to learn and practise baby care, hence KMC helped them to be better prepared to take care of their baby when the baby is discharged. They also felt that they received extra attention from the healthcare staff who commended their efforts to practise KMC.

*“Which mother wouldn’t want to be with their baby. Every mother will want it (KMC) when given the chance.”* (Mother 1042).

*“I got to know my baby can’t suck properly (while doing KMC). The physiotherapist came and taught me how to stimulate him with mouth massages.”* (Mother 1103).

*“Each time when they (nurses) pass by, they will ask: “Are you doing ok? How’s the baby doing?”… reviewing us 3 to 4 times within an hour …. they will also come and look at how my baby had latched on my breast during feeds.”* (Mother 1042).

Participants mentioned that KMC allowed fathers to have an opportunity to be involved in the care of their baby and it was a form of family activity.

*“I don’t mind doing it (KMC). My wife couldn’t do it all the time. We take turns.”* (Father 1202 whose wife was undergoing the traditional 4-week post-partum confinement practices in a traditional Chinese post-partum centre).

*“My husband … is even more enthusiastic than me. … he will do KMC when I am not around.” (Mother 1103)*.

*“I am willing to try KMC (with my husband) now for our three babies. We will be closer than ever as parents.”* (Mother 1204 of a set of triplets).

*“I intend to continue this at home. I will teach my mother-in-law and husband to do it at home when I return to work.”* (Mother 1028).

Only two participants knew that KMC could be practised by fathers. Others were either unsure or did not think that KMC could be practised by men.

*“I don’t think they (fathers) are allowed to do KMC. I don’t think the nurses will make such arrangements for them. When fathers are around, their presence will cause a lot of inconveniences.”* (Mother 1028).

*“My husband has asked me before if he is allowed to do KMC. I wasn’t sure. If fathers are allowed, they are actually giving us support.”* (Mother 1030).

All participants reported that they felt ‘exposed’ and ‘naked’ when they were doing KMC because they would have to reveal themselves more than they would normally do in public, particularly when they had to expose their chest when placing their baby on their chest or when they were taking the baby out of the KMC tube.

*“The place is so open. Men could just be behind the screen. Sometimes when the ward is crowded, the screens can accidentally be pushed, opening up a gap for everyone to see me doing KMC behind the screen).”* (Mother 1143).

##### Cognition

Participants reported that KC returned a sense of parenthood to them. KMC was seen as an opportunity to be with their baby, which was missed when they were separated by medical care.

*“…while doing KMC, I finally felt like a mother. I have longed to hold him.”* (Mother 1030).

*“How would I describe what I feel… well, I feel a sense of motherhood because when I put him on my body, I gave him my finger and he grabbed it.”* (Mother 1042)

All participants perceived that both parent and baby benefitted from KMC. This included better weight gain for their baby, stronger parent-baby bonding, and earlier discharge.

*“It’s amazing to see him gaining weight so fast. He sleeps very well too…”* (Mother 1028).

*“I have wondered how my baby’s weight can be increased faster … and how he can go home earlier…. After doing KMC, (pause) my babies’ weight has increased. We are all allowed to go home tomorrow.”* (Mother 1444 of a set of twins).

During the interview, all participants mentioned that they wanted to get their baby home as soon as possible. They felt that feeding the baby is of great importance because they have been told that their baby would only be discharged upon achieving a particular weight.

*“We all want to go home earlier. We want our babies to come home with us. But I can’t. My baby’s weight is still not enough. I need to feed my twins more.”* (Mother 1444 of a set of twins).

Therefore, many of them placed more importance on spending their time expressing breast milk to stock up their milk supply instead of spending time performing KMC as they felt that the former is crucial to earlier discharge.

*“I want to do it (KMC) but I just don’t have the time because it’s time to express my breast milk now. I don’t have much milk, so I need to spend more time expressing milk. And I am too tired for KMC after I have finished expressing my milk.”* (Mother 1143).

*“I have seen some mothers who are too exhausted to do KMC (after milk expression and self-care).”* (Mother 1103).

Participants had the perception that KMC should not be done during and immediately after a feed, thus narrowing the window opportunity for KMC.

*“I don’t dare to do KMC when I know my baby was just fed or still feeding. I am afraid he will vomit. I have to wait. But not long after, my baby needs another feed again, so I have no opportunity to do it.”* (Father 1202).

*“I was stopped by the nurse when I arrived (to do KMC). My baby had just finished his feeding and he needed his rest first. She told me to do it later. But it’s so hard to find time because my baby needs a feed every 2–3 hours.”* (Mother 1028).

All participants were also concerned that they might be in the way of medical staff who were attending to their baby if they performed KMC. They were hesitant to seek help or ask the healthcare staff questions about KMC for fear of disturbing them.

*“I just sit in that area (the KMC corner), and just talk to him (the baby) there. … I feel that I might be disturbing the doctors or something like that when I am doing KMC. The nurses won’t be able to feed my baby either.”* (Mother 1103).

*“I didn’t approach any doctors. Doctors are very busy.“* (Mother 1028).

#### Facilitating factors

Participants reported receiving ‘special privileges’ in the wards when they practised KMC. These ‘privileges’ ranged from being on the priority list to room-in, use hospital services such as screens and front button blouses, and be given extra attention and easier access to their baby.

*“Before this, I was commuting between my house and hospital. While I was doing this (KMC), the nurses reserved a bed for me and told me to freely use the portable screens for privacy.”* (Mother 1103).

We also found participants mention repeatedly that they felt there was a lack of facilities to practise KMC in the wards.

*“There is only a two-seater sofa in the KMC and breastfeeding corner …… frequently occupied with mothers breastfeeding their babies so there is no place to do KMC.”* (Mother 1028).

Participants mentioned that they would like comfortable chairs and sufficient clean laundered KMC tubes so they had a clean one to use for each subsequent KMC.

*“I couldn’t do it (KMC). The KMC tube was still wet.”* (Father 1202 who only had one KMC tube and it had just been washed).

*“ To do it (KMC), I have to change my clothing and wear this thing (KMC tube) and I find this a nuisance. I am afraid that my milk will leak out and soil the KMC tube. Washing the used KMC tube is another issue. … there’s no proper place to keep things clean and dry. … lots of cockroaches roaming around here…. when I tell them (healthcare staff) …, nothing is done.”* (Mother 1103).

They also requested an in-ward multi-user breast pump. One mother who had brought her own breast pump to the ward faced difficulties in cleaning, drying and storing her breast pump. They thought with the pump, milk expression time could be shortened and they would have more time to practise KMC.

In addition, participants felt that support from healthcare staff was sometimes lacking.

*“It’s the staff here… they are difficult. They chided me because I was not doing it correctly and disallowed me to hold my babies for long.* *The nurses warned me that my babies will be clingy if I keep doing KMC. The nurses also made me feel like I am hindering them from being able to tube feed my babies when they are doing KMC with me.”* (Mother 1204 of a set of triplets).

*“I approached a nurse for help but she told me that she was busy and (she) asked me to seek help from other nurses. When I went to the other nurses, they said that it was not their responsibility to help me because they were not the nurse in charge of caring for the baby at that point in time”* (Mother 1028).

Participants also reported that educational materials on KMC were inadequate

*“There are many posters in the room but I don’t notice any on KMC.”* (Mother 1030).

## Discussion

Using the Triandis’ Theory of Interpersonal Behaviour, the likelihood of practicing KMC is a function of the ‘habit’, ‘intention’ and the ‘facilitatory or inhibitory conditions’ to this practice. Each of these variables provided us with a deeper understanding of the barriers and enablers to the practice of KMC are.

### Habit

‘Habit’ according to the Triandis model refers to how automatically an action is practised. Our study revealed that KMC was not something automatic that parents would practise in our hospital settings. It was not a norm for our parents because KMC awareness was not present for almost all our participants. This was similar to findings in two other studies where parents would not have thought about asking for an opportunity to practise KMC unless this was being offered by healthcare staff [14, 15]. In fact, even after they were subsequently introduced to the KMC practices, we found that they considered KMC as the least important part of care during their baby’s hospitalisation. To them, the ‘norm’ or ‘habit’ was to expect that babies warded in hospitals needed constant medical attention and treatment, and that incubator care was better than KMC care. This is similar to a study by Charpak who found that parents considered KMC sub-standard care [16].

Of note, for participants of Chinese ethnicity, it was a cultural norm to ensure adequate post-partum rest [17]. They perceived that KMC interfered with this norm because the mother could not ‘rest’ when she was practicing KMC. This was made worse when she had to travel from her home or confinement centre to the hospital frequently due to a lack of rooming-in facilities at the hospital.

If parents could be informed about KMC earlier, it would give them more time to comprehend what is to be expected, and perhaps be able to accept this as part of their norm [18, 19]. A good time to start KMC introduction would be during the antenatal period before the birth of the baby [18, 20] so that there is already an awareness of what KMC is about. Introducing KMC during the antenatal period is problematic when it is offered only to preterm infants since preterm birth is often unexpected. However now that the recommendation is to offer it to all infants and that it be started immediately after birth, it is time to incorporate it into all antenatal education packages [4, 5]. Subsequently, the importance of KMC should be re-emphasised when the baby is born, similar to what is currently being done with antenatal breastfeeding education. There is a lot of evidence suggesting that KMC uptake is better when parents are made aware of their roles earlier [14, 21].

### Intention

Regardless of whether or not KMC is an established habit, the Triandis model outlined a complex interplay of three variables that influences a person’s intention to actually practise KMC: (1) the affect component (the emotions felt when KMC is practised), (2) the social component (social attitudes towards the appropriateness for our parents to practise KMC and (3) the cognitive component (the value given to the perceived consequences of practising KMC). Depending on the circumstances, these three variables can interact in various ways, resulting in them being either an enabler or a barrier to KMC practices [13, 22].

#### Affect

There was a variety of affect experienced by our participants, some of which increased their intention to practise KMC and some which discouraged them from practising it.

It was interesting to note that all participants started off with negative emotions such as fear and embarrassment. In general, almost all participants were caught unaware and unprepared to have such close bodily contact with their baby. Many studies have reported how people have perceived direct skin-to-skin contact and the kangaroo position to be unusual or improper [14, 16]. They were also embarrassed and uneasy because they felt ‘exposed’ to strangers while learning or practising KMC in the ward. Many also started off uncertain and fearful of harming their baby with KMC. However, all our participants described how their affect evolved to more positive emotions over time. Many of them described the joy and fulfillment they felt after trying out KMC a few times; and some mentioned that they wished they had started KMC earlier. This emotional change phenomenon was also described by Lemmen et al. [14].

It was also common to have conflicting affect occurring at the same time for many parents.

For example, they felt valued to have a role in the care of their baby in the hospital setting as it returned them a sense of parenthood instead of being a stranger to their baby; and perceived that their baby enjoyed and thrived with KMC (positive affect). At the same time, they felt that KMC was challenging and demanding because they were not ready to shoulder the responsibility of monitoring their baby’s well-being throughout the practice. To them, KMC was part of the medical management, and they were worried about making mistakes that could result in untoward consequences to their baby (negative affect). There are several other studies that have similarly reported how parents appreciated the opportunity brought about by KMC to participate in the care of their baby but at the same time were stressed by challenges faced during KMC [20, 21].

For participants that roomed-in with their baby at the hospital and practised KMC in their room, they had conflicting feelings when they were told that they had to practise KMC for at least 6 hours a day. While they appreciated the 6 hours of quality time they could spend with their baby as they practised KMC, they also felt lonely and isolated from their family because family members were not allowed to go into the mothers’ room. Therefore, if they were doing KMC when family members came to visit during the limited visiting hours, it would mean that they do not get to meet their family. They did not feel comfortable enough to go out to the common visitor’s area when they were practising KMC because KMC was perceived to be something that should be done in private. The above affect has been described by Charpak in two of her studies [16, 23]. Our participants also felt pressurized when healthcare staff continued to emphasise that they had to practise KMC for at least six hours a day to be able to bring beneficial effects to their baby. To them, six hours of KMC was very difficult to achieve, and some of them felt demotivated when they were unable to achieve the targeted hours.

Therefore, perhaps better communication between healthcare staff and parents could facilitate a better uptake of KMC [24]. Healthcare staff could reduce parental stress and anxiety by assuring them that monitoring the baby during KMC would be a joint effort between healthcare staff and the parent; and that even if the 6-hour duration was not achieved, doing the minimum 1-hour duration of KMC would still bring benefits to both the baby and the parent. Changing the ‘visitation’ policy to be more family-centred could also help [25]. There is increasing data showing that family-centred care and flexible visitation at intensive care unit settings motivate parents to continue with KMC [21, 26, 27].

These steps would also help to reduce the sense of being isolated in confined spaces when they are practising KMC.

#### Social

Socio-cultural norms have an important influence in parents’ intention to practise KMC. We found that one of the main reasons why our participants continued to practise KMC despite the many barriers was because there was a social expectation for them to do the best for the baby. They felt obliged to practise KMC because it was considered to be beneficial for their baby. KMC also allowed fathers to participate in caring for the baby, thus for some of our participants, it was seen to be a family activity that would bond the family. In addition, they also perceived that healthcare staff was more approving of parents who practised KMC as staff paid more attention to their baby if they came to practise KMC.

However, in Malaysia where it is generally not socially acceptable to have physical contact in public, most of our participants were uncomfortable with practising KMC in the ward. This was especially so for Islamic parents who perceived they could be censured socially for inappropriate exposure of their body. For example, to ensure that the baby’s head is visible, they would need to expose part of their upper chest which would normally be covered. Our male participant in turn had guilty feelings that it might be embarrassing for female healthcare staff who had to help him position his baby on his bare chest.

Another social factor in Malaysia that had an influence on the uptake of KMC is that women are generally considered the main caregivers for infants. Therefore, there was an expectation that KMC would only be performed by mothers, a finding similar to Lydon 2018 [20]. That could be the reason why there was only one father doing KMC during the study period. In contrast to a recent report from Scandinavia [28], we found that the concept that men could also practise KMC was not considered by most participants and even some healthcare staff. KMC by fathers or surrogates have also been reported to be under-utilised in the neonatal intensive care unit (NICU) [29, 30]. This is not surprising given that the word ‘mother’ is part of the practice’s name, and that more people are familiar with the term ‘Kangaroo Mother Care’ rather than just ‘Kangaroo Care’ [31]. Historically, the implementation of KMC started with only the involvement of the baby’s mother, and most studies have focused on maternal kangaroo care. Opportunities for fathers to participate in their baby’s care are also less compared to the opportunities given to the baby’s mother [32]. This is something that should be addressed because regardless of whether KMC is practised by the baby’s mother or father, the benefits are similar [33] or at most marginally better with the mother [34].

In other settings, where the socio-cultural norms differ, other factors play a bigger role in parents’ intention to practise KMC. For example, in places where preterm births are considered a stigma [14, 21, 35, 36] or where domestic responsibilities at home were a big priority [26, 35], parents did not want to be seen practising KMC. However, this phenomenon was not present among our participants and was not a barrier to KMC practices here.

Meanwhile, it was obvious from our participants that peer-to-peer support was not available in our setting. KMC was also not part of social conversations and none of our participants had talked about KMC with their family members or with other parents in the ward. Introducing peer support groups would be an area to explore. Support from peers, local communities and religious leaders have been reported to be important for KMC uptake [18]. In addition, it would be useful to encourage the involvement of the extended family as this is a social norm in Asian communities. Family-centred care could be a strategy for scaling up parental involvement and building up parents’ confidence [5, 37].

#### Cognition

As described above, a lot of thought went through the minds of parents when they were trying to decide whether or not to practise KMC; particularly around how they felt when practising KMC, how socially acceptable was the practice, and whether KMC brought benefits or harms to their baby.

All participants in this study had experienced the benefits of KMC not only for their baby but also for themselves and their family. The main benefits they perceived were faster weight gain, improved bonding, earlier discharge, and better skills in handling their preterm newborn. These benefits of practising KMC were similarly found in several other studies or reviews [2, 24, 38] and were strong motivators for parents to practise KMC. In this study, the earlier discharge was particularly of interest because all our participants had a strong desire to have their baby discharged as soon as possible and return to some degree of normality.

A phenomenon unique to our study was that all our participants were very focused on milk expression. Their understanding from healthcare staff was that they had to express as much breast milk as possible to have sufficient feeds for their baby so that their baby would gain sufficient weight to be discharged. Although our participants perceived that their baby’s weight gain improved with KMC, they were worried that KMC could rob them of the time needed to express breast milk for their baby. As the WHO has recently updated its guidelines to promote milk expression while in the kangaroo care position, we are optimistic that a shift in mindset will occur in the near future [5, 39].

Despite the evidence that KMC does improve weight gain for preterm infants, many of our participants perceived that the reason behind their baby’s weight gain was from the breast milk intake and that KMC did not contribute to this. Furthermore, our participants had understood from the healthcare staff that KMC should be avoided during and immediately after feeding. As their babies were fed every two- to three-hourly, this left the participants a very small window of time to practise KMC. We hope that with the updated WHO guidelines, there will be a shift in awareness that KMC can continue when the baby is being fed [4, 39].

On top of that, KMC was perceived by our participants as something that would get in the way of healthcare staff and obstruct them from carrying out medical management of their baby. They also did not feel free to ask questions regarding KMC for fear of disturbing the healthcare staff who were thought to be very busy all the time. All these thoughts could have resulted in them being hesitant about practicing KMC because they perceived that it could delay the discharge of their baby from the hospital. As this phenomenon was not reported in other KMC-related studies, a possible explanation might be because our participants were recruited from the hospital setting and the other studies had participants from the community where discharge was not a factor for consideration.

If healthcare staff could help parents understand much earlier that KMC actually improves breast milk production and increases the success of prolonged direct breastfeeding for preterm infants [40, 41], mothers would feel less pressure to spend their time expressing breast milk. Instead, they would be motivated to spend more time on KMC which in turn could naturally increase breastfeeding success. Even in cases when feeding expressed milk via nasogastric tube is necessary, the semi-inclined position during KMC should help to reduce the incidence of regurgitation or vomiting. By promoting the early initiation of KMC as a means of breastfeeding support immediately after birth, the provision of breast contact and colostrum within the first hour of life is likely to be more successful.

Of note, another reason for parents’ hesitancy to practise KMC could be because they were not cognisant of the consequences of not practicing it. This is comparable to vaccine hesitancy which often is due to the lack of perception of the consequences of not vaccinating [42, 43]. Our participants were more concerned about adhering to traditional postpartum routines because they believed that failure to do so would result in health problems for the mother in the future [17]. Some of the practices needed for these traditional routines would make the mother think twice about performing KMC. Such practices included the need to keep her body fully covered to avoid draughts, hence she would be reluctant to expose herself while placing her baby skin-to-skin on her.

### Facilitating conditions

In the Triandis model, ‘facilitating conditions’ are to be considered in addition to habit and intention when predicting the uptake of KMC practice. Our study revealed several facilitating factors that were already in place which helped them with KMC. This included the availability of a dedicated KMC corner for them to practise KMC in the ward. However, our participants felt that much could be done to improve this space. For one, it was too small and cramped. In addition, they did not feel confident that the portable screens or curtained cordoned-off space were good enough for their privacy. This was because these structures were mobile and hence could accidentally be moved, and they would then be in full view of people in the ward at any moment. Lack of privacy and space for KMC has also been reported in several studies, including a systematic review [24]. This is a difficult barrier to address in our setting, and the permanent provision of privacy and space for KMC was a facilitating condition that our participants felt was necessary. If space could be made available, the provision of fixed partitions, private rooms and family rooms would be an important facilitating factor [5, 6].

The availability of rooming-in facilities was also a facilitating factor. Unfortunately, there were very limited beds for mothers to room-in. Mothers who were unable to room-in with their baby experienced additional difficulties as they had to travel to and from the hospital. They would also have to time their travel to meet the visiting hours and meet the narrow window of time between feeds to be able to practise KMC. For those who had a car, finding a parking space in the hospital compound was also expressed as a difficulty. All of these were factors preventing parents from being successful in providing KMC for the recommended six hours a day. There is an urgency in addressing this problem because the latest WHO guideline update in November 2022 recommended that the duration of KMC should be increased to at least eight hours a day.

However, what was considered ‘facilitating conditions’ could also differ as some of our participants had different individual preferences. For example, most of our participants expressed their appreciation for the availability of the KMC tube in the ward. The KMC tube eased the practice of KMC, alleviating the physical strain on parents who no longer need to maintain constant vigilance holding on to their baby. Utilizing the KMC tube also enhanced parental mobility and afforded them the freedom to use their hands to engage in various everyday activities like reading [5]. All these made them feel that the time spent doing KMC was more productive. Unfortunately, none of our participants were aware that wearing the tube was optional and not essential for KMC. For participants who did not like to wear the tube or for those that did not have extra tubes to change into when their tube became soiled, the tube became a barrier and not a facilitator. This highlighted the importance of better communication between healthcare staff and parents to avoid misunderstanding the purpose of these facilities.

### Strengths of the study

We believe we had achieved theoretical sufficiency with our cohort of participants because a considerable amount of information given by these participants was similar to one another. Using the Triandis model as a framework strengthened our analysis and allowed us to be more certain about the factors behind the practice. Although we could not find other studies which used this model, we found literature that had used other grounded theories, developed either by the investigators themselves [44] or using Andersen’s model (Andersen and Newman Framework of Health Services Utilization) [38]. The similarities of variables in the Triandis and Anderson models will allow comparison of findings in our study with findings from studies using the Anderson model to see if the results are generalisable and enable more applied research based on psychosocial theories, thus providing more practical intervention programmes. However, before interventions are planned, it would be important to understand the perspective of others in the same context, most importantly the perspective of healthcare staff. We are in the process of analysing data from staff in the same setting and plan to report this in a separate paper.

### Limitations

Although there was no new information obtained by the time we reached the eighth participant, there was only one participant of the male gender. While some of the information provided by our sole father was also mentioned by the mothers we recruited, there may be information that only fathers could provide that we were unable to gather. For example, the male participant expressed embarrassment when female staff had to assist him in positioning the baby. We do not know whether other fathers may have felt the same. The lack of male key informants could be because men have not been playing an active role as KMC providers and this in turn might be due to cultural norms.

We also did not interview parents who were not practising KMC, hence might be missing information on other barriers our participants had not brought up. This is important because it could conceivably be a reason for the low uptake described in the introduction. This study also did not have participants that were practising KMC at home. Future studies may consider including these to provide a more complete picture. Further studies can also be done to seek the views of parents who were introduced to KMC but did not practice it so that we understand factors behind why they decided not to do it.

## Conclusions

A deeper understanding of the factors influencing the uptake of KMC using the Triandis behavioural model has provided a way forward to help improve its uptake and sustainability in our setting. While barriers such as overcrowding in the wards may be a major challenge for us and in other resource-limited settings, earlier preparation of parents for KMC with provision of appropriate information during the antenatal period is something that could be addressed relatively easily and immediately. Once in place, this could allow other enablers to be strengthened. This includes better healthcare and peer-to-peer support; and improved communications and visiting hours. Further studies can then be done to see if these will result in improved uptake and sustainability of KMC both in the hospital and in the community settings.

### Electronic supplementary material

Below is the link to the electronic supplementary material.

Supplementary Material 1

Supplementary Material 2

## Data Availability

The transcripts are available from the corresponding author on reasonable request.

## Abbreviations

IPA: International Paediatric Association
KMC: Kangaroo Mother Care
MREC: Medical Research & Ethics Committee
NICU: Neonatal Intensive Care Unit
SEA-URCHIN: South East Asia- Using Research for Change in Neonatal Infection
WHO: World Health Organisation
