# Supplementary material for: Exploring factors influencing the uptake of kangaroo mother care: key informant interviews with parents
Source: BMC Pregnancy Childbirth. 2023 Oct 3;23:706. doi: 10.1186/s12884-023-06021-6 (PMC10548712; doi:10.1186/s12884-023-06021-6)
Supplement: Supplementary file 1 — Supplementary Material 1 [file 12884_2023_6021_MOESM1_ESM.pdf]

Appendix 1: Interview questions for parents

1. Have you done KMC before? If yes, tell me what was it like?
    - What did you find good about KMC?
    - Was there anything you were concerned about? Please share.
2. If you were to talk to another parent about KMC, what would you tell him/her?
3. What are some of the things that would prevent a parent from practising KMC for at least 6 hours a day?
4. Can you give some suggestions on how to make your KMC experience better?
