# Supplementary material for: Exploring factors influencing the uptake of kangaroo mother care: key informant interviews with parents
Source: BMC Pregnancy Childbirth. 2023 Oct 3;23:706. doi: 10.1186/s12884-023-06021-6 (PMC10548712; doi:10.1186/s12884-023-06021-6)
Supplement: Supplementary file 2 — Supplementary Material 2 [file 12884_2023_6021_MOESM2_ESM.pdf]

Appendix 2 Table 1

**Baseline characteristics**	**Range**
Parent’s age (years)	24 to 38
Parent’s gender<br>Male<br>Female	 1<br>8
Singleton<br>Twins<br>Triplets	7<br>1<br>1
Baby’s birth weight (g)	1240 to 1800
Baby’s gestation (weeks)	27 to 33
Duration of hospital stay prior to the interview (days)	15 to 69
Duration of NICU stay prior to the interview (days)	2 to 60
